# Simultaneous Bilateral Fracture Dislocation of the Talus: A Case Report

**DOI:** 10.5812/traumamon.11228

**Published:** 2013-08-11

**Authors:** Mohammad Hosein Taraz-Jamshidi, Omid Shapari, Reza Shiravani, Saeed Moalemi, Ali Birjandinejad

**Affiliations:** 1 Orthopedic Research Center, Shahid Kamyab Hospital, Department of Orthopedic Surgery, Mashhad University of Medical Sciences, Mashhad, IR Iran

**Keywords:** Dislocation, Talus, Fracture Fixation, Internal

## Abstract

**Introduction:**

Fracture - dislocations of the talus are typically due to high energy injuries. Displaced fracture - dislocations of the talus have poor outcomes in general and complications are common. Although talar fracture is common and comprises the second most common tarsal fracture, bilateral fracture - dislocations of the talus are rare. Not many reports regarding the subject can be found in the literature.

**Case Presentation:**

We report a patient with bilateral fracture - dislocations of the talus treated by open reduction and internal fixation. This patient was a 25 year-old man who sustained bilateral fracture - dislocation of the talus due to a motor vehicle accident.

**Conclusions:**

Bilateral talar fracture - dislocation is rare. The surgical approach discussed together with the pathomechanics of this injury can yield good short term results.

## 1. Introduction

Despite the fact that talus fractures constitute the second most common fractures involving the tarsal bones, bilateral fracture-dislocations of the talus can be considered rare and few cases have been reported (1, 2).The appropriate treatment suggested by most experts includes permanent anatomical reduction (3). Even with appropriate treatments, fracture-dislocations of the talus have poor prognoses (2, 4), and may be associated with complications such as avascular necrosis and posttraumatic arthritis in both subtalar and tibiotalar joints (5). Soft tissue injuries are commonly seen in association with this fracture-dislocation. Emergency reduction is our top priority since it reduces the risk of developing soft tissue injuries and postoperative complications (2, 6-8). Simultaneous fractures of neck and body of the talus are rare and are thought to have serious outcomes. Intraarticular injuries are ominous complications and may lead to articular surface disruption at the talus neck. Also, due to the fact that fracture-dislocations of the talus are a result of high-energy impacts, these injuries are often seen in patients with multiple traumas. All these points should be taken into consideration while managing the patient. Open fracture-dislocations of the talus are treated urgently (5, 9).

## 2. Case Presentation

**Figure 1.**
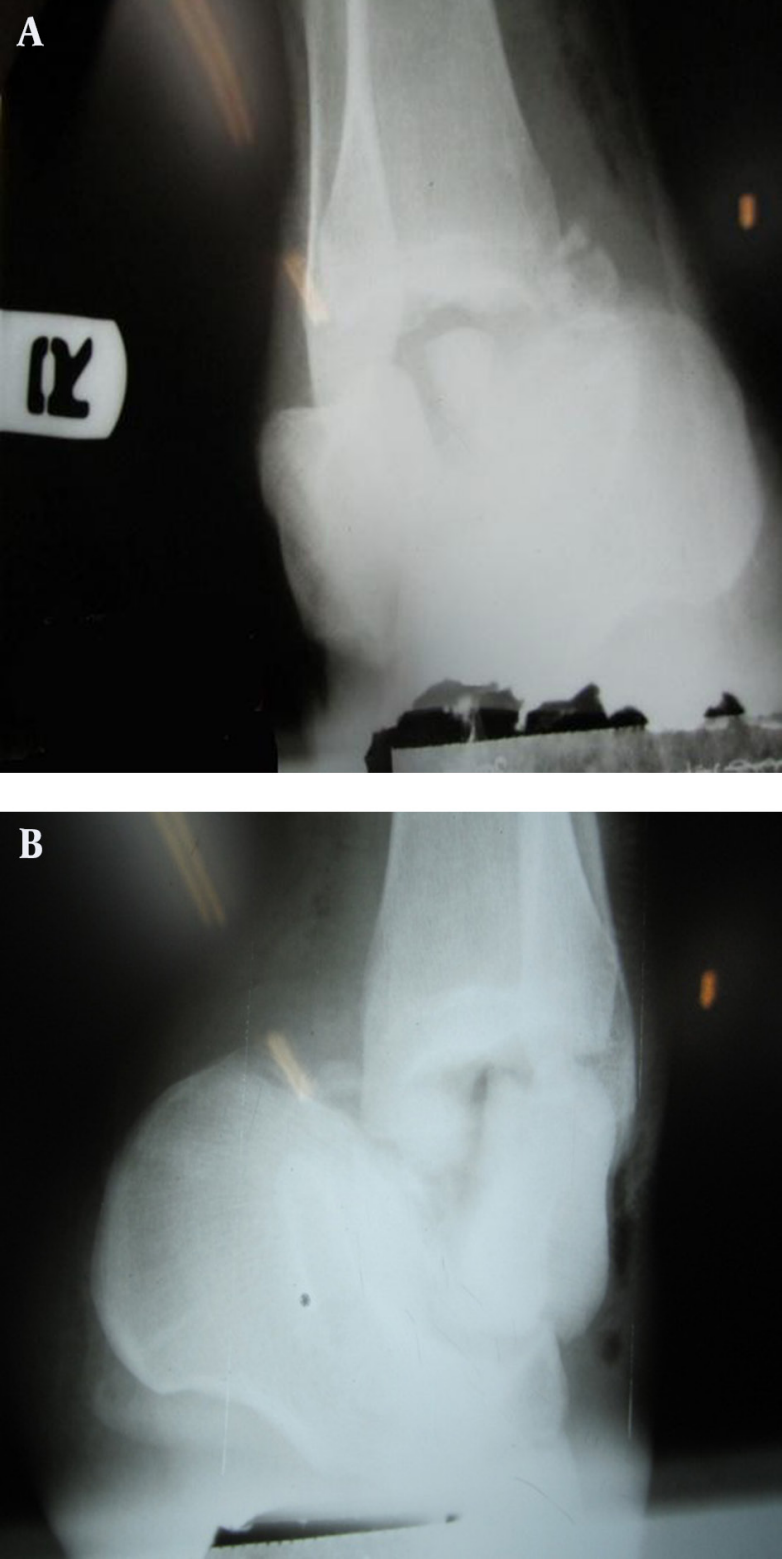
Left Talus Neck Fracture With Subtalar and Talonavicular Dislocation, A. Comminuted Fracture of Right Talus Body and Lateral Process With Subtalar and Talonavicular Dislocation, B.

**Figure 2.**
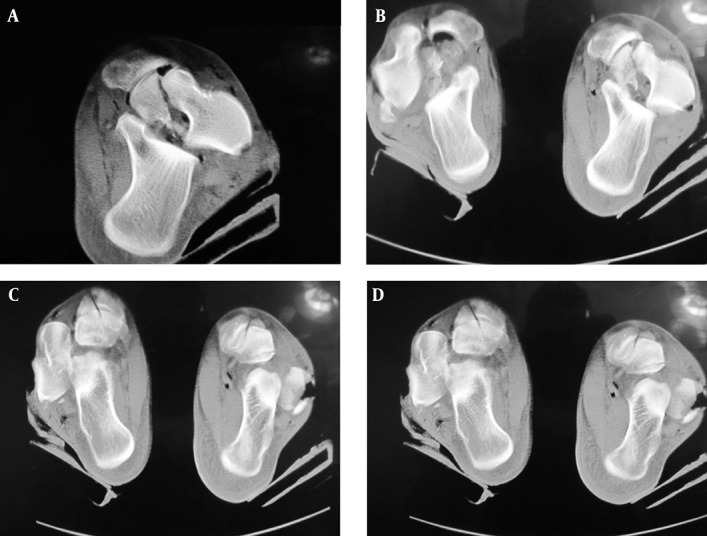
Ankle CT Scan of The Patient With Bilateral Talar Fracture-Dislocation

A 25 year-old young man was injured in a motor vehicle accident (inability to control the vehicle due to violation of the speed limit). When his car reached the U-turn, it slid off the road and flipped over. Possible mechanism of injury was dorsiflexion pronation of the left side and dorsiflexion supination of the right side. The patient was brought in conscious and hemodynamically stable at the time of admission; he presented with severe bilateral ankle swelling, numerous bilateral ecchymoses and blisters without any open wounds. On physical exam, toe circulation was mildly delayed but the extremities were warm and no evidence of ischemia could be found. In the systemic review, no serious head, spinal or abdominal trauma requiring emergent measures could be noted. Ankle and plantar radiographs were obtained which demonstrated left ankle Hawkin type 4 open talar neck fracture with subtalar and talonavicular dislocation and a 3 cm open wound (Figure 1 A). On the right side, Hawkin type 4 open and comminuted fracture of the right talar body and lateral process with subtalar and talonavicular dislocation, and 3 cm open wound was noted (Figure 1 B, Figure 2). In spinal radiography there were T10 and T11 vertebral body fractures.

**Figure 3.**
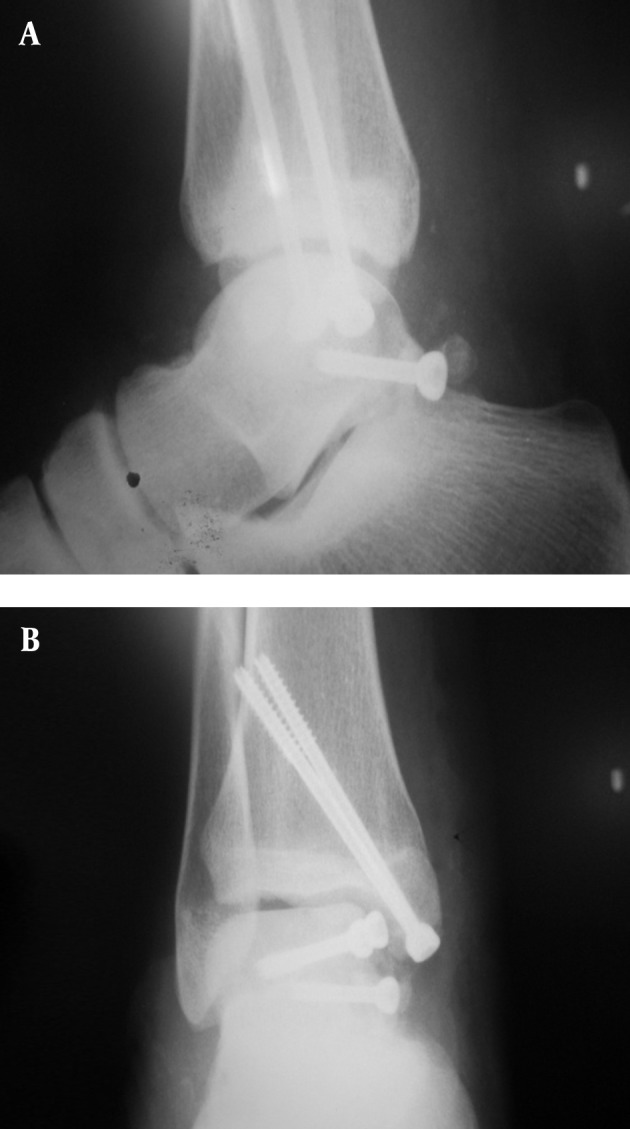
Internal Fixation of Right Talus Body and Posterior Medial Tubercle

**Figure 4.**
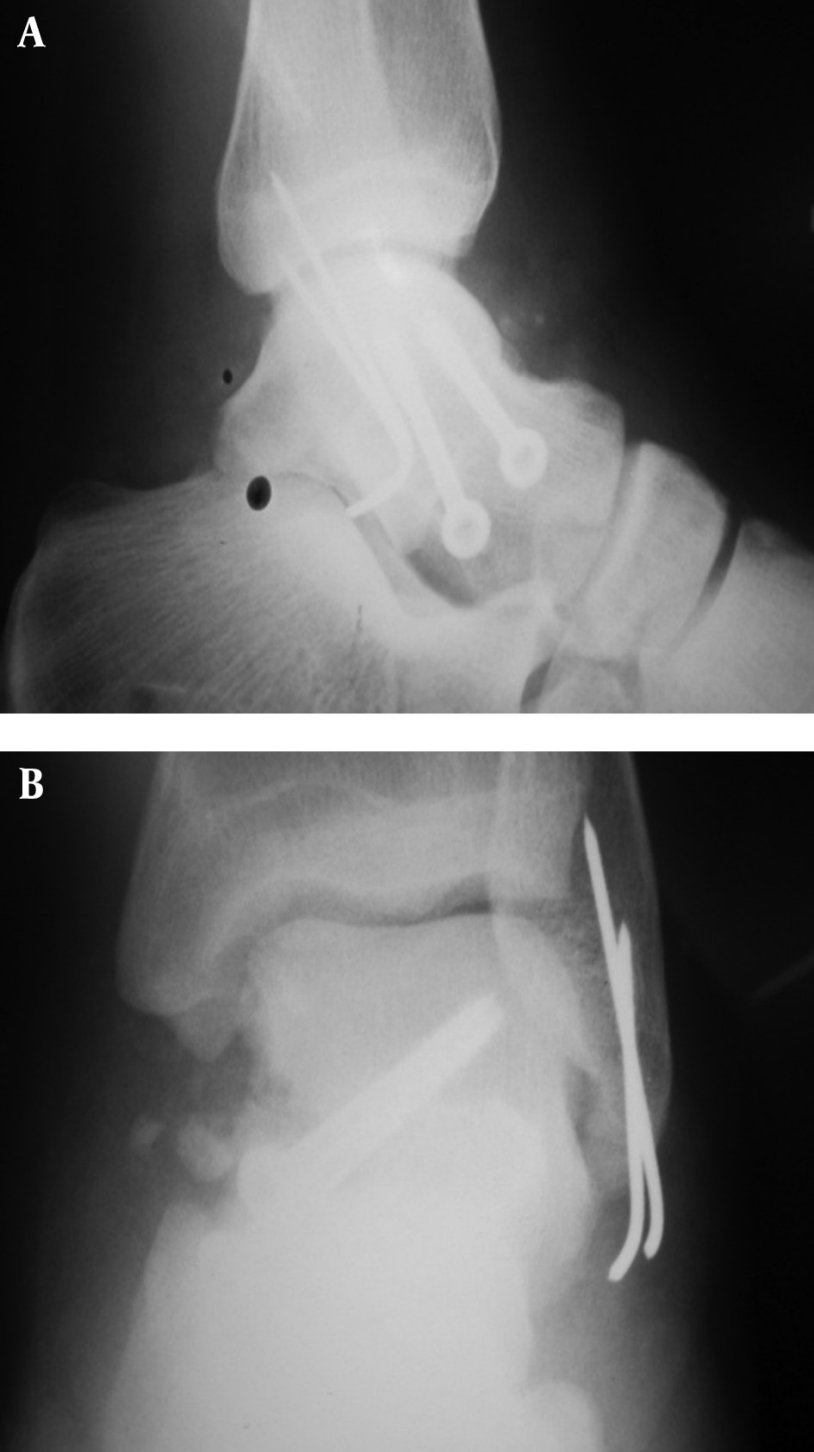
Internal Fixation of Left Talus Neck with Medial Wall Comminution and Left Lateral Malleolar Avulsion

**Figure 5.**
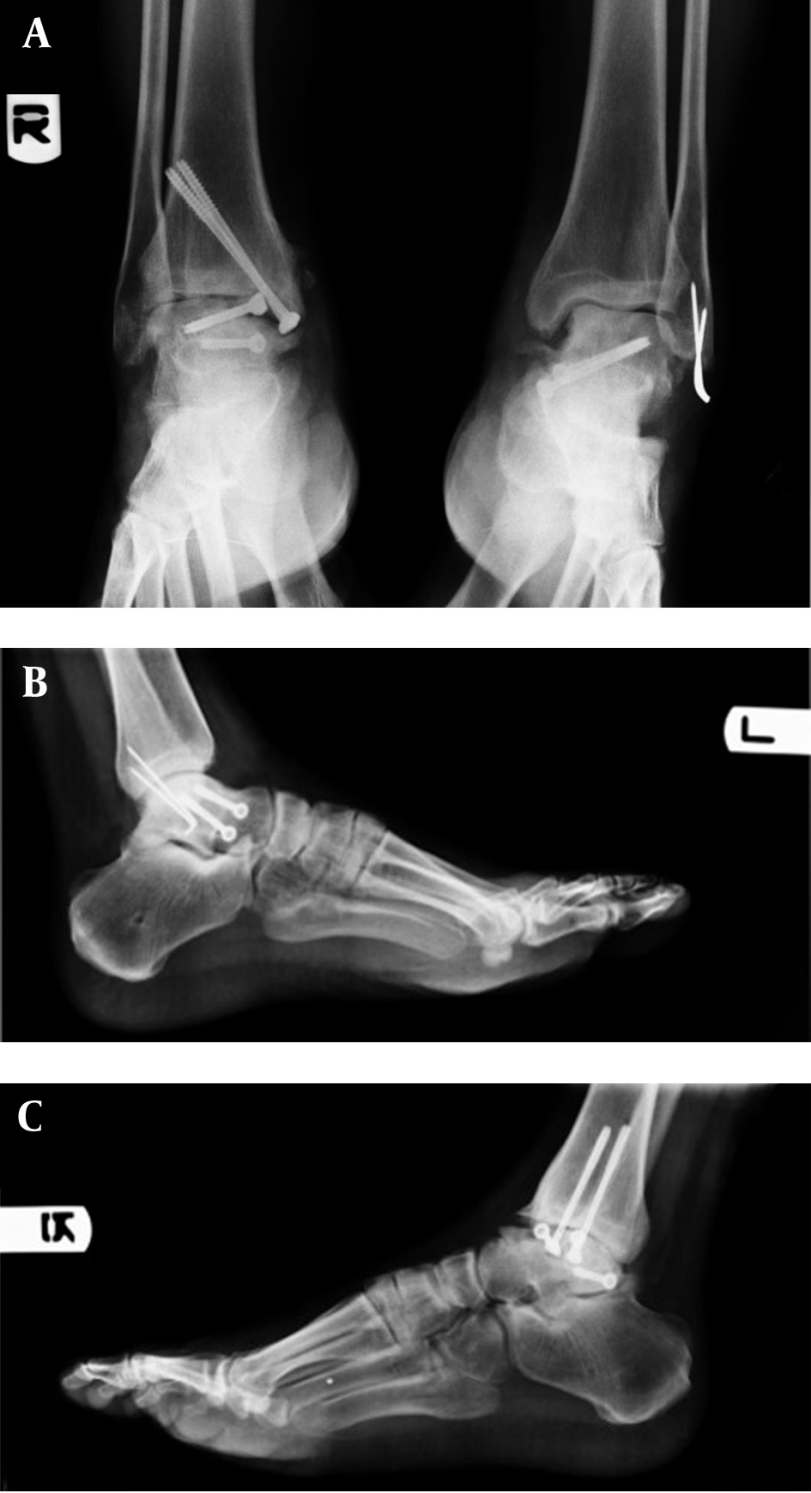
AP and Lateral X-Ray of Right and Left Ankle After Two Years Follow Up

In the right lower extremity, multiple fragmented talar body fracture and posterior medial tubercle were opened via an anteromedial approach. Chevron osteotomy of the medial malleolus was performed. After intraarticular irrigation and minimal soft tissue manipulation, reduction was performed by three partial thread cannulated and cancellous screws (4 mm). Consequent intraarticular irrigation was performed and the location of malleolar osteotomy was fixed by 2 malleolar pins (Figure 3 A, B). In the left extremity, the talus neck fracture with medial wall comminution and lateral malleolar tip avulsion was opened by simultaneous anteromedial and lateral approach for a better view, after which intraarticular irrigation and debridement took place. Neck reduction was performed using 2 cannulated cancellous screws (4 mm), and lateral Malleolus fracture was fixed using two pins(number 1.1) (Figure 4 A, B). Antibiotic prophylaxis was continued up to 48 hours after the operation. The patient was discharged with short splints on both lower extremities without any wound complications. The patient’s condition after 2 years follow up was 20 degrees plantar flexion, zero dorsiflexion with moderate pain and AVN and collapse in talar body on the right side. The Foot and Ankle Disability Index (FADI) score was 58/7 and 40 degrees plantar flexion 20 dorsiflexion no pains no AVN on the left side. The Foot and Ankle Disability Index (FADI) score was 82/7 (Figure 5). The patient`s quality of life was good based on the SF-36 score.

## 3. Conclusions

Overall 2 % of all lower extremity injuries and 5-7% of foot injuries are associated with talar fractures. Talar fracture is one of the common fractures of the tarsal bone and often includes talus head and neck. On the contrary, simultaneous talar body fracture and dislocation can be considered pretty uncommon (2). Falling and axial compression force are the most common mechanisms responsible for talar body fractures (1, 10, 11). Talar neck and head fractures are much more common when compared to talar body fractures (9, 12), and most result from dorsiflexion and talar neck being driven into tibial plafond (13). Few reports of bilateral talar fracture have been reported up to now (13). According to Hawkin, talar neck fracture-dislocation can be classified into 4 types (2, 4, 9):

Type I: Fracture with no displacement

Type II: Subtalar + Tibiotalar dislocations

Type III: Subtalar dislocation

Type IV:Subtalar + Tibiotalar + Talonavicular Dislocation (although very uncommon, it seems that the reported number is an underestimation of its incidence, probably due to missed diagnoses)

In a case of simultaneous talar body fracture-dislocation reported by Sayegh et al. in 2009, it was claimed that no similar case has ever been reported and published in UK. A 29 year-old patient with low socioeconomic status was admitted with talar body fracture and talonavicular and subtalar dislocation. Due to wound infection, the injury had been fixed by K-wire. In the follow up, the patient reportedly developed posttraumatic arthritis but did not show any signs of AVN. Ankle ROM was decreased on both sides but the patient's overall activity after treatment had been satisfactory (3). It has been suggested that in cases of talar body fracture with posterior dislocation (Types III and IV in Hawkin's Classification) urgent reduction be performed since they may be accompanied by severe soft tissue injury, rapidly progressive edema, and necrosis. Due to the aforementioned accompanying complications, closed reduction is not a plausible option. Recommended surgical treatment includes anteromedial approach with sufficient muscle relaxation for reduction, accomplished by applying traction and plantar flexion. To achieve complete reduction, a lateral approach and internal fixation is used eventually (9). In our literature review, we found only one report on simultaneous bilateral fracture-dislocation of the talus since 2009 (discussed above). The present case provides opportunity for further investigation of the clinical and therapeutic aspects regarding this condition. This case report also enables us to study the possible postoperative complications and the needed care (5, 9).Our patient had left talar neck, lateral process and right talar body fractures with bilateral subtalar and talonavicular dislocations. He underwent urgent bilateral internal fixation. Most talar fractures have poor postoperative prognoses. In most patients investigated by numerous studies, certain postoperative disabilities have been reported and the patients are known to have certain degrees of permanent pain and limitation in movement. Besides, short term complications such as infection, increased risk of AVN, and secondary osteoarthritis can be regarded as inevitable after-effects of this fracture (3, 13-17). Considering short term and long term complications, the postoperative outcomes and the complications seen in this bilateral fracture-dislocation discussed here hold importance and may help to elucidate some of the details regarding this subject.
